# Tonic and phasic effects of reward on the pupil: implications for locus coeruleus function

**DOI:** 10.1098/rspb.2022.1545

**Published:** 2022-09-14

**Authors:** Laura Cole, Stafford Lightman, Rosie Clark, Iain D. Gilchrist

**Affiliations:** ^1^ Henry Wellcome Laboratories for Integrative Neuroscience and Endocrinology, Bristol Medical School, University of Bristol, Bristol, UK; ^2^ School of Psychological Science, University of Bristol, 12a Priory Road, Bristol BS8 1TU, UK

**Keywords:** pupil, locus coeruleus, reward, auditory oddball paradigm

## Abstract

The locus coeruleus (LC), a nucleus in the pons of the brainstem, plays a significant role in attention and cognitive control. Here, we use an adapted auditory oddball paradigm and measured the pupil dilation response, to provide a marker of LC activity in humans. In Experiment 1, we show event-related pupil responses to rare auditory events which were further elevated by task relevant. In Experiment 2, by asking participants to silently count the number of oddballs, we demonstrated that the task-relevance elevation was not a result of the generation or execution of the manual response. In Experiment 3, we observed two separate effects of reward on the pupil response. First, we found an overall increase in pupil area in the high compared to the low-reward blocks: a sustained effect reminiscent of the tonic changes that occur in LC. Second, we found elevated event-related pupil responses to behaviourally relevant stimuli in the high-reward condition compared with the low-reward condition, consistent with phasic changes in LC in response to a stimulus. These results highlight the complexity of the relationship between the pupil response and reward, and the inferred role of LC in both top-down and bottom-up cognitive control.

## 1. Introduction

The locus coeruleus (LC) is a brainstem nucleus, located in the dorsal pons; it is the primary source of norepinephrine in the CNS and the sole source in the cortex [1]. It plays a central role in modulating arousal and attention both of which are essential to efficient goal-directed behaviour, influenced by both the bottom-up salience of sensory events and the top-down goals of the animal. However, investigating the LC in humans directly is extremely challenging. Non-invasive imaging techniques such as magnetic resonance imaging (MRI) are hampered by the LC's small size, the presence of strong physiological noise, the proximity of surrounding brainstem nuclei [2] and large individual difference in location and size [3–5].

One alternative, non-invasive but indirect technique to investigate the human LC is to measure pupil dilation. Electrophysiological recordings from primate animal studies indicate that under certain circumstances, pupil size fluctuates with LC activity [6–8]. LC activity is characterized by short-burst phasic responses, evoked by a stimulus and sustained low-amplitude tonic responses [9]. The pupil response can also take two forms: a tonic baseline component, where the pupil changes in size for a sustained period of time, and a phasic response, which is an event-related transient change [10].

The phasic responses can be evoked by salient stimuli. For example, in an auditory *oddball task*, participants are presented with an infrequent tone stimulus among a train of more frequently presented alternative tones. This infrequent oddball stimulus has been shown in humans to both evoke a transient pupil dilatory responses [2,11–13] and increase the fMRI BOLD response in the vicinity of the LC [2].

The range of salient stimuli that have been used to evoke these phasic responses is quite broad. However, perhaps the shared characteristic of these stimuli is low probability—such stimuli are salient because they tend to stand out in comparison with more frequently presented, expected stimuli. So, as stimulus probability decreases, saliency increases, and so does the phasic response [14]. Similar findings have been reported in animal studies of LC activity, albeit with a phasic response both to low-probability target and non-target salient stimuli [15,16].

The phasic LC response may not be exclusively selective for behaviourally relevant events, but may also occur for low-probability non-relevant stimuli, perhaps because these unexpected stimuli generate a related involuntary behavioural orienting response in a bottom-up manner [17]. Some studies have reported habituated phasic responses to trains of target stimuli, suggesting that task relevance may not be as important to the phasic LC and pupil response as salience determined by low probability [6,18]. However, others have reported higher amplitude pupil responses to target stimuli even when they have a higher probability than distractor stimuli [19]. It is unclear whether task relevance is necessary for the LC and pupil phasic response or whether the response can result from a bottom-up stimuli alone [19,20].

In most cases, a target is only a target (and so is salient in a top-down manner) when there is a response required to that stimulus. As a result, in most laboratory tasks, in both humans and animals, the target stimulus is temporally confounded with a motor response to the target so that the phasic response is at least as likely to be related to behaviour rather than sensory processing [21]. It is unclear whether an overt behavioural response is required to evoke a phasic change in LC activity or pupil size, or whether behavioural significance alone is sufficient.

In animal studies of LC function, the training and maintenance of performance is directly dependent on the task including an explicit reward protocol and reward plays a pivotal role in adaptive gain theory [9] discussed in more detail below. Despite this, the majority of studies of the human pupil response, and in particular those using an oddball paradigm, have not included an explicit reward schedule. Reward incentives constitute a direct modulation of task relevance and the requirement for top-down control. That is, more highly rewarded stimuli are likely to compete more strongly for attentional resources [22–25]. Recent studies have implicated the LC in such intrinsic (or top-down) motivation. For example, Bouret & Richmond [26] found that reward cues modulated phasic LC responses, with larger responses for cues that signalled larger rewards. Pupillometry studies have also reported associations between reward stimuli (incentive cues and reward delivery) and both phasic and tonic changes in pupil size [11,27,28]. Some evidence that supports a relationship between reward and pupil size comes from studies using techniques in which the reward delivery is paired with a reward cue or behaviour [29–31]. The ‘liking’ effect that occurs with reward delivery is dissociable from incentive salience or wanting a reward, which is an effect induced by the presentation of a reward cue. In fact, these two effects have been found to activate distinct neuronal circuits [32]. Most reward studies have focused on the dopaminergic system, but the above evidence opens a novel avenue of investigation, as it indicates that the LC, which is a major norepinephrine nucleus, also has a role in motivation and reward.

The present study uses an auditory oddball paradigm to address three questions: First, what is the contribution of stimulus probability and task relevance to the pupil response (Experiment 1)? Second, are the phasic pupillary responses seen to task-relevant stimuli in Experiment 1 a result of the preparation or execution of a manual response (Experiment 2)? And third, what is the effect of reward on the pupillary response (and LC activity by proxy)?

## 2. General method

All participants had normal or corrected-to-normal vision and were paid for participation. Informed participant consent was obtained for all studies, and all were approved by the Faculty of Health Sciences Research Ethics Committee, University of Bristol, UK. Participants were recruited from the University of Bristol community via poster advertisements, webpage and email lists.

All experiments were written using Psychophysics Toolbox v3 [33] running under Matlab 2015a (The MathWorks, Natick). The computer display was a ViewPixx 22.5-inch liquid crystal display screen with a spatial resolution of 1920 × 1200 pixels (VPixx Technologies); viewing distance was 60 cm. Pupil area (left pupil) and eye position was recorded at a sampling rate of 500 Hz by a tower-mounted Eyelink 1000 (SR Research, Canada) eye tracker, which has a typical operating spatial resolution of 0.5°. Head movement was minimized using a chin and forehead rest. Manual responses were recorded via a computer keyboard. Testing was carried out in the windowless testing laboratory with very limited ambient light other than that arising from the display. There were no light point sources in the participant's field of view other than the screen. Ambient illumination measured at the eye position during a trial was 21.2 lx (RS-92 Light Meter, RS Group PLC, UK).

Participants were asked to carry out an auditory oddball task. In the task, participants were played a sequence of short tones (100 ms) and required to listen for, and respond to, a rarely occurring tone (the oddball target, 8% of tones). The sequence of tones also included another rarely occurring tone (deviant tone, 8% of tones) and a frequently occurring tone (standard tone, 84% of tones), neither of which required a response. Tone order was completely randomized within a block. The standard tone always had a frequency of 1400 Hz, and the oddball and deviant were either 1200 or 1600 Hz (counterbalanced within or between participants, depending on the experiment), and the inter-tone interval was jittered between 1.2 and 1.7 s. Participants were instructed to maintain fixation on a white fixation cross that was present at the centre of a grey screen throughout each block of trials. Each testing session was made up of a practice block and experimental blocks. Errors were defined as making manual responses to the deviant or standard stimulus, or not responding to the oddball stimulus. These trials were excluded from the pupil analysis as were participants who made more than 20% errors overall.

The raw pupil data was subjected to the following pre-processing. We coded as ‘missing’ data where the eye tracker detected a blink using the default Eyelink setting. Around these detected blinks, there were additional periods of instability in the recorded pupil size. We coded as ‘missing’ pupil data 200 ms either side of the blink onset and offset. The recorded pupil size also depends on the eye position and so we coded as ‘missing’ all pupil samples where the eye position was 2 degrees of visual angle away from the central fixation point. We then carried out a linear interpolation across the missing data based on the first and the last recorded pupil measurement. We next carried out a linear detrending of the data across the duration of the block of trials as alertness is correlated with pupil size [34]. Using a number of different artificial pupils, which were laser printed on paper, we converted the arbitrary units of pupil size produced by the Eyelink eye tracker into pupil size in mm^2^.

For each trial (tone), we extracted the pupil size data time-locked to the tones, from 500 ms before the tone onset to 4000 ms after the tone onset. To account for individual, and trial-by-trial, difference in the position of the peak response, and to capture the peak response to the stimulus across all three experiments with a common averaging window, we averaged the pupil response in each individual trial across two time-based epochs [35]. The first was the *pre-stimulus baseline epoch* (−500–0 ms) and the second was the *stimulus response epoch* (500–2000 ms). For each trial, we then subtracted these two epochs to give our primary dependent measure: *change in pupil response*. For Experiment 3, we also carried out an analysis of the pre-stimulus baseline epoch.

We used linear mixed effects modelling (LMM) to explore trial-by-trial changes in pupil size using the JASP software [36]. A model with both varying slopes and intercepts on the random effects was fitted in all cases unless there was evidence based on model Akaike information criterion that a simpler model was more appropriate [37]. We used sum contrast coding as this makes the interpretation of interactions easier; however, the model parameters are not as straightforwardly linked to a particular level of a factor. As a result, we report, and plot, estimated marginal means with 95% confidence intervals for each condition and factor and test specific contrasts with Holm–Bonferroni correction. For ease of reading, reporting of these models is kept rather brief but full details of the models along with the raw data and analysis scripts are available at (https://osf.io/s2qkc/).

## 3. Experiment 1

### (a) Methods

There were 27 participants. Three participants were excluded because they made more than 20% manual response errors on the task, resulting in a final sample of 24 participants (15 females; age range 18–69).

Participants carried out one practice block of 25 trials followed by two experimental blocks of 250 trials. In one block, the high tone was defined as the oddball, and in the other the low tone was defined as the oddball. Block order was counterbalanced between participants. Participants were instructed to make a manual response to the oddball tone.

### (b) Results

**Figure 1.**
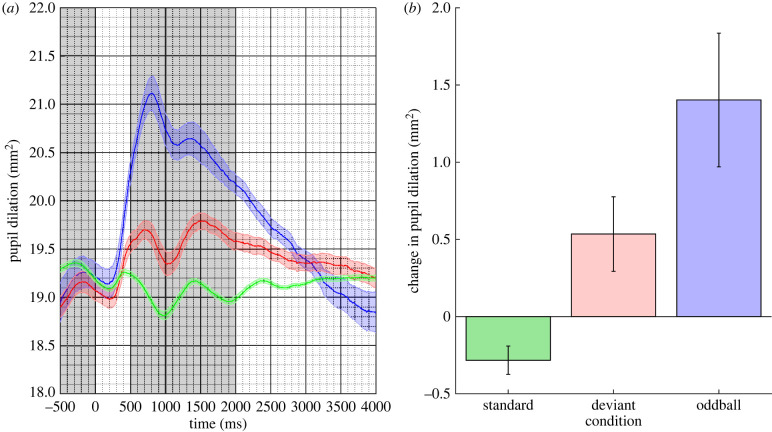
(*a*) Mean pupil responses to the standard (in green), deviant (in red) and oddball (in blue) stimuli for Experiment 1. The coloured shaded regions represent the standard error of the mean corrected for within-subject and condition variance. (*b*) The estimated marginal mean difference in pupil response between the pre-stimulus baseline and stimulus response epochs (shaded in grey in (*a*)) for the three conditions. Error bars are 95% confidence intervals. (Online version in colour.)

Mean pupil responses in the three conditions are plotted in figure 1. The figure shows a clear response to the stimulus onset for the oddball trial type alongside a smaller response to the deviant when compared with the standard trial type.

A LMM was fitted to the *change in pupil response* data with *Participant* as a random effect, and *trial type* (oddball, deviant and standard) as a nominal fixed effect. The estimated marginal means for the three conditions are plotted in figure 1 and were oddball: 1.403 mm^2^; 95% CI [0.970, 1.836]; deviant: 0.535 mm^2^; 95% CI [0.293, 0.776] and standard: −0.283 mm^2^; 95% CI [−0.374, −0.191]. The difference between the standard and the oddball was reliable (mean difference: 1.686 mm^2^; *z* = 6.602, *p* < 0.01) as was the difference between the standard and the deviant (mean difference: 0.817 mm^2^; *z* = 5.323, *p* < 0.01).

In addition, we note that the 95% CI for the standard is outside, and below, zero. This suggests that, in this condition, there was a reliable reduction in pupil size in response to the tone consistent with some degree of habituation or active suppression.

### (c) Discussion

We compared the pupil response with three classes of auditory tones: a frequently occurring standard tone; an infrequent deviant tone which did not require a response; and an infrequent oddball tone that participants were instructed to make a manual response to. Both the oddball and the deviant were perceptually salient in that they were rarely occurring stimuli and distinct from the standard tone. Both stimuli produced a phasic pupil response that was time-locked to the stimuli onset [20]. The oddball and the deviant tones were equally rare and because they were counterbalanced were equally perceptually salient. The difference between these two stimuli was that participants were instructed to make a response to the oddball. The task-relevant oddball produced a larger phasic response when compared with the task-irrelevant deviant, suggesting that the pupil response is modulated by top-down factors above and beyond perceptual salience [19].

We have so far argued that the current experiment demonstrates a task-relevance effect on the pupil response. However, as discussed in the introduction, it remains possible that elevated pupil response to the oddball stimulus is a result of the motor preparation or motor execution associated with the manual response. To investigate this, in Experiment 2, we adjusted the oddball task by adding a counting block, in which participants were asked to silently count oddball stimulus presentations. In this condition, no manual response is required but the oddball stimulus remains task relevant. We compared this directly with a condition that is a direct replication of Experiment 1, where the participants are required to manually respond to each oddball as before.

## 4. Experiment 2

### (a) Methods

There were 25 participants. Five participants were excluded for producing error rates of 20% or more in one or more experimental blocks, or due to missing pupil data, resulting in a final sample of 20 participants (14 females; age range 18–29).

We manipulated response type within the auditory oddball paradigm to investigate the effects on pupil area. Participants were either asked to silently count the number of oddball stimuli and to state the total count at the end of the block (counting response type); or participants made a manual response to each oddball stimulus (manual response type). Participants carried out one practice block of 25 trials followed by four experimental blocks of 125 trials, the two blocks for each response type were administered consecutively and counterbalanced across participants. The oddball tone was also counterbalanced across participants so that the low tone was the oddball stimulus for half of the participants, and the high tone was the oddball for the others. The procedure was the same as Experiment 1, with the exception that between the second and third blocks participants were reminded that the task rules had changed.

### (b) Results

**Figure 2.**
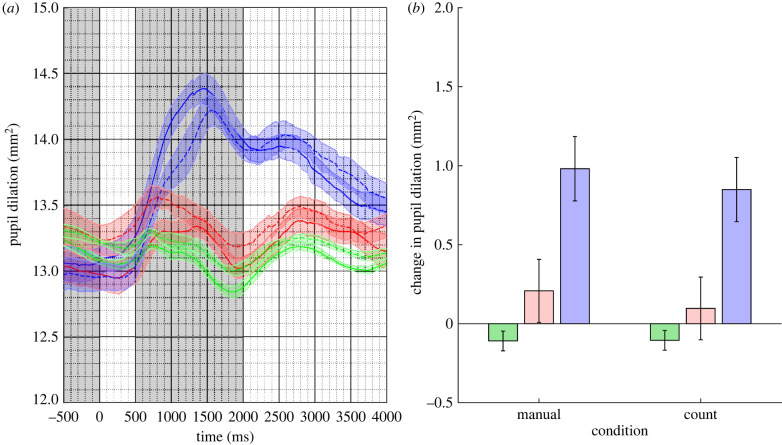
(*a*) Mean pupil responses to the oddball (in blue), deviant (in red) and standard (in blue) stimuli for the counting (dotted lines) and manual (solid lines) response conditions in Experiment 2. The coloured shaded regions represent the standard error of the mean corrected for within-subject and condition variance. (*b*) The estimated marginal mean difference in pupil response between the pre-stimulus baseline and stimulus response epochs (shaded in grey in (*a*)) for the counting and manual conditions. Error bars are 95% confidence intervals. (Online version in colour.)

Mean pupil responses are plotted in figure 2. As in Experiment 1, the figure shows a large pupil response to the oddball stimulus, with smaller responses to the deviant and standard stimuli. This pattern is clear for both response types.

A LMM was fitted to the *change in pupil response* data with *trial type* (oddball, deviant and standard) and *response type* (manual and counting) as nominal fixed effect. *Participant* was fitted as a random effect with random slopes on trial type only. The slope parameter estimates for trial type were reliable (both *p*'s < 0.001) but not for response type (*t*_9749_ = −1.639, *p* = 0.101); nor was there any evidence of any interactions. The 95% CIs on the estimated marginal means for the manual: 0.360 mm^2^; 95% CI [0.266, 0.454] and the counting: 0.281 mm^2^; 95% CI [0.186, 0.373] conditions show considerable overlap (mean difference: 0.080 mm^2^; *z* = 1.639, *p* = 0.101).

We did not find evidence that the response type lead to a change in stimulus-induced pupil dilation.

### (c) Discussion

The results of the current experiment replicate those in Experiment 1. The oddball stimulus results in the largest pupil response compared with either the baseline or the deviant and this effect is present whether there is a manual response required to the oddball or not. This suggests that the effect of the oddball stimulus on the pupil is not a result of motor preparation or execution, but of task relevance. While these findings are in line with the previous investigations that have reported task-evoked pupil dilations in the absence of overt behavioural responses, there has been no direct comparison of pupil responses with targets in the presence and absence of a motor event [38,39]. Our results provide a direct comparison of pupil responses in the presence and absence of motor preparation or output.

In comparison with Experiment 1, the pupil response to the oddball and deviant stimuli is slightly delayed. As the manual response condition involved the same task paradigm as before, this increased latency of the phasic response is probably due to individual differences in pupil response latency within the participant groups.

Our results suggest that pupil size is sensitive to task relevance. The task relevance of behaviourally significant stimuli can be modulated by the reward associated with that response. In other words, reward processes can modulate the salience of a stimulus in a top-down manner. Precisely how these reward-based top-down effects compete or interact to influence cognition and behaviour is unclear. Although there is some conflicting evidence, one study found that stimuli associated with greater rewards receive more attention than behaviourally relevant stimuli associated with low reward [23]. Although we expect a manipulation of reward to influence task performance either via the response time or error rate (e.g. [40]), it is unclear if this will affect the pupil response and if it does, whether this will be a phasic or tonic effect. We are especially interested in this notion given the evidence that reward incentives evoke LC activity as well as changes in pupil size [41]. In Experiment 3, we investigated the relationship between pupil size and reward in our oddball paradigm.

## 5. Experiment 3

### (a) Methods

There were 25 participants. Five participants were excluded for producing error rates above 20%, or due to missing pupil data, so the final sample contained 20 participants (12 females; age range 19–45).

We repeated the paradigm from Experiment 1 as before, but we manipulated the monetary reward participants received using a lottery incentive reward scheme [42], which was explained to the participants at the start of the experiment. Participants carried out one practice block of 25 trials followed by four experimental blocks of 125 trials. In two of the experimental blocks, the oddball stimulus was associated with 20p; in the other two, it was associated with £20. Onscreen instructions conveyed the amount of reward associated with the oddball stimulus prior to each block, and participants were instructed to make quick manual responses to the oddball stimulus if they wanted to have a chance of winning the associated reward. We added visual stimuli to the task to provide participants with visual feedback: if participants responded in time to the oddball stimulus (less than 0.7 s), *£00.20* (in low-reward blocks) or *£20.00* (in high-reward blocks) appeared onscreen within a fixation box. If participants did not respond in time, *£00.00* was displayed instead. Visual stimuli made up of the same number of pixels were presented within (*£XX.XX*) and between (*£++.++*) each trial so that there was no difference in luminance throughout the task and importantly, between trials. The inclusion of these reward stimuli extended the trial duration when compared to Experiments 1 and 2 but trial duration was constant across all conditions within the experiment.

At the end of the task, one of the oddball trials from the whole task was selected at random, and if the participant had responded within 0.7 s to the selected oddball stimulus, they received the associated monetary reward in cash. Two high- and two low-reward blocks were alternated within participants, and the block order was counterbalanced across participants. Half of the participants were told to respond to the low tone and the other half were told to respond to the high tone.

### (b) Results

**Figure 3.**
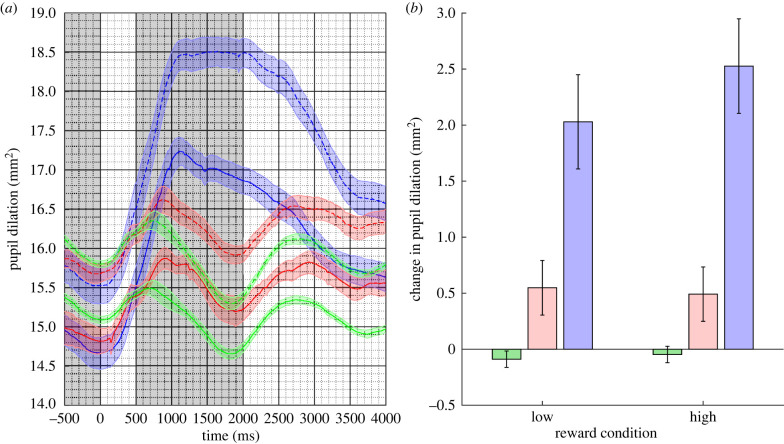
(*a*) Mean pupil responses to the oddball (in blue), deviant (in red) and standard (in blue) stimuli for the high-reward (dotted lines) and low-reward (solid lines) conditions in Experiment 3. The coloured shaded regions represent the standard error of the mean corrected for within-subject and condition variance. (*b*) The estimated marginal mean difference in pupil response between the pre-stimulus baseline and stimulus response epochs (shaded in grey in (*a*)) for the high- and low-reward conditions. Error bars are 95% confidence intervals. (Online version in colour.)

Mean pupil responses are plotted in figure 3. As before, there is a clear pupil response to the oddball stimulus and smaller responses to the deviant and standard stimuli, respectively. This pattern is clear for both reward conditions.

A LMM was fitted to the *change in pupil response* data with *trial type* (oddball, deviant and standard) and *reward condition* (low and high) as nominal fixed effect. *Participant* was fitted as a random effect with random slopes on trial type only. The slope parameter estimates for trial type were reliable (both *p*'s < 0.001) and for reward condition (*t*_8559_ =−2.627, *p* = 0.009), and there was evidence for an interaction (*t*_8708_ = 1.780, p = 0.075 and *t*_8954_ = −3.454, *p* < 0.01 for the two slopes). The 95% CIs on the estimated marginal means are plotted in figure 3 and appear in table 1. A test of the difference in the pupil response between the standard and the oddball comparing the low- and the high-reward conditions was reliable (mean: 0.456 mm^2^; *z* = 3.281, *p* = 0.002) but not for the same comparison between the standard and deviant conditions (mean −0.099 mm^2^. *z* = −0.767, *p* = 0.443). This suggests that the presence of the higher reward results in a larger pupil response to the stimuli in the oddball condition but that this effect is not present in the deviant condition. This is evidence of a modulation of the phasic pupil response to the increased reward that is specific to the task-relevant stimuli.

**Table 1.** Estimated marginal means for Experiment 3.

trial type	reward condition	95% CI		
		estimate	lower	upper
standard	low	−0.089	−0.162	−0.016
oddball	low	2.029	1.609	2.450
deviant	low	0.549	0.305	0.792
standard	high	−0.047	−0.120	0.026
oddball	high	2.527	2.105	2.949
deviant	high	0.492	0.249	0.734

To investigate the effect of reward condition on pupil area within the pre-stimulus epoch, we carried out a separate LMM with *reward condition* (low and high) as a nominal fixed effect and *participant* as a random effect. The estimated marginal means for the two conditions were low: 15.135 mm^2^; 95% CI [12.636, 17.634]; high: 15.866 mm^2^; 95% CI [13.358, 18.375]. A test of the difference in the pupil response between the low- and the high-reward conditions was reliable (mean: 0.732 mm^2^; *z* = 2.444, *p* = 0.015).

These results indicate that the pupil was generally more dilated in the high-reward condition at baseline and are evidence of a tonic effect on the pupil of the higher reward.

Mean response times to the oddball stimulus in the high-reward condition were 670 ms as compared with 690 ms in the low-reward condition. This effect was reliable: *F*_1,19_ = 6.09, *p* = 0.023, partial ETA squared = 0.243; indicating that the increased reward also speeded up manual response, consistent with an increase in motivation.

### (c) Discussion

Our results demonstrate an amplification of the phasic pupil effect by reward, as well as a tonic effect of reward condition.

This effect of reward condition on pupil size is likely related to motivation or ‘wanting’ reward, rather than receiving reward, as reward delivery occurred after task completion. Previous work has suggested that extrinsic motivation can influence top-down salience to bias attentional selection in favour of reward incentives [23]. Therefore, the phasic and tonic reward effects on the pupil could signify an effect of target relevance on the pupil, driven by motivation or wanting the reward. The phasic reward effect is stimulus locked and represents an interaction between top-down task relevance and extrinsic motivation, resulting in a magnified pupil response in comparison with a change in pupil size that is evoked by task relevance alone. This suggests that these factors can combine to amplify the pupil response further so that a target stimulus produces an even larger response if they are linked to higher monetary reward. By contrast, the tonic effect of reward on the pupil reflects a block-wide effect of reward condition that is not stimulus locked and therefore constitutes an effect of reward condition in the absence of task relevance. Similar effects have been reported by Koelewijn *et al*. [43], investigating the effects of monetary reward using speech stimuli.

The effect of the reward manipulation on the reaction times to the oddball target confirm that the reward manipulation is effective and that it results in a change in overt behaviour that this correlated to the corresponding changes in pupil response.

## 6. General discussion

In Experiment 1, we investigated whether the phasic pupil response would differ between three trial types, which varied in perceptual salience (stimulus probability) and task relevance. We demonstrated that both factors result in a stimulus-locked pupil response. The results from the standard stimuli suggest that the pupil appears to be relatively insensitive to the frequently occurring and non-task-relevant events. In Experiment 2, we demonstrated that the task relevancy effect is still present when no overt response is required to each stimulus. This suggests that the stimulus-related pupil response is not a result of the preparation or execution of an overt response. Finally, in Experiment 3, we investigated the effect of reward on the pupil response and found significantly greater tonic and phasic pupil dilations in the high-reward condition. This suggests that reward incentives can both amplify the effect of task relevance on the pupil response as well as result in an overall dilation of the pupil because of the reward context.

Pupil responses are a well-established indirect marker of LC activity. Our findings suggest that the LC is sensitive to both, bottom-up, perceptual salience as well as top-down task relevance including the modulation of task relevance by reward. This highlights the importance of behavioural significance for task-evoked LC responses and provides support for theories of LC function that postulate that this, rather than bottom-up salience, drives the response [44].

Theories of LC function propose that phasic LC firing serves as an interrupt signal or a ‘network reset’, which is followed by a reorganization of brain networks and corresponding behavioural reorientation in favour of environmental contingencies [45,46]. Our findings provide support for this framework: we found a phasic pupil response to the behaviourally relevant oddball stimulus. However, we also consistently observed a phasic pupil response related to the deviant stimulus, which was not behaviourally relevant. This effect of task-irrelevant salience on the pupil might suggest a role for the LC as an alerting signal responding to unexpected, and so potentially dangerous or threatening events within the environment in a relatively automatic manner [22]. This role would also explain why studies have found that phasic LC responses to stimulus-driven salience may habituate: although physically salient stimuli automatically capture attention they can be discarded if repeated exposure reveals that they do not constitute a threat or possess behavioural significance. This fits with the experimental data that bursts of LC firing evoked by stimulus-driven salience eventually habituate to facilitate appropriate attentional control [47] and with data that optogenetic activation of LC-TH+ neurons results in novelty-associated memory enhancement [48]. This is not to say that the LC does not respond and contribute to attentional and behavioural orienting towards a behaviourally significant event, but that it functions in the attentional control of both types of salience (bottom-up and top-down), rather than only behavioural relevance. This notion is also supported by a recent animal study, which reported that attentional control is modulated by two distinct projections to the prefrontal cortex originating in the LC [49].

One influential theory of LC function is adaptive gain theory [9]. Within this theory, LC switches between a phasic and a tonic responding mode. The phasic response is selective to task-relevant stimuli and not distractors, even if they are infrequent, and the two modes correspond to a switch between an exploitation to an exploration state for the organism. Our results support the suggestion for these two modes, but in Experiment 3, we see evidence that the two modes can operate concurrently. In addition, in Experiment 1, we present evidence for a phasic pupil response to task-irrelevant infrequent distractors although this may correspond to a more general task relevance associated with a potential threat that is signalled by infrequent events. Finally, although it is tempting to suggest that the increased reward in Experiment 3 leads to an increased tendency to remain in an exploiting mode, our experiment does not allow for change in task (and exploration) to seek increased reward (although see [50]).

The present study used pupillometry to investigate human LC function, as the close coupling between this structure and the pupil has been well documented. However, the underlying neural pathway linking the LC with the pupil is unclear. There is some evidence to support a direct connection between the LC and the pupil, such as single-cell recordings of LC neurons in monkeys, which have demonstrated short bursts of LC activity coinciding with pupil dilation [51]. There is also a commonly cited animal study that reported an association between tonic pupil size and LC activity; however, this study has never been published [52]. Perhaps the most convincing evidence comes from Joshi *et al*. [7], who found that salient auditory tones evoked transient pupil dilations as well as neuronal activity in the LC, inferior colliculus and the anterior cingulate cortex in monkeys. The amplitude of the pupil response was only related to the amplitude of neuronal activity in the LC, and not any other recorded sites. What's more, direct stimulation of the LC evoked reliable transient pupil dilations. This evidence demonstrates a causal relationship between LC activity and pupil size.

The distinction between stimulus-driven and goal-related processes is an important one because they involve different underlying neural mechanisms. For example, stimulus-driven attentional control activates the temporoparietal lobe, and detects events based on physical features or stimulus-driven characteristics, such as physical salience, novelty and unexpectedness [25]. By contrast, goal-related control activates the intraparietal and superior frontal lobes, and selects stimuli based on certain cognitive factors, such as goals, motivation and expectations [24]. Here, we show that the pupil—and therefore we argue the LC—is sensitive to both.

## Data Availability

The data and analysis scripts are available at https://osf.io/s2qkc/ and are publicly available.
